# Quality of Systematic Reviews of Observational Nontherapeutic Studies

**Published:** 2010-10-15

**Authors:** Tatyana Shamliyan, Robert L. Kane, Stacy Jansen

**Affiliations:** Minnesota Evidence-based Practice Center, University of Minnesota School of Public Health; University of Minnesota School of Public Health, Minneapolis, Minnesota; University of Minnesota School of Public Health, Minneapolis, Minnesota

## Abstract

**Introduction:**

High-quality epidemiologic research is essential in reducing chronic diseases. We analyzed the quality of systematic reviews of observational nontherapeutic studies.

**Methods:**

We searched several databases for systematic reviews of observational nontherapeutic studies that examined the prevalence of or risk factors for chronic diseases and were published in core clinical journals from 1966 through June 2008. We analyzed the quality of such reviews by using prespecified criteria and internal quality evaluation of the included studies.

**Results:**

Of the 145 systematic reviews we found, fewer than half met each quality criterion; 49% reported study flow, 27% assessed gray literature, 2% abstracted sponsorship of individual studies, and none abstracted the disclosure of conflict of interest by the authors of individual studies. Planned, formal internal quality evaluation of included studies was reported in 37% of systematic reviews. The journal of publication, topic of review, sponsorship, and conflict of interest were not associated with better quality. Odds of formal internal quality evaluation (odds ratio [OR], 1.10 per year; 95% confidence interval [CI], 1.02-1.19) and either planned, formal internal quality evaluation or abstraction of quality criteria of included studies (OR, 1.17 per year; 95% CI, 1.08-1.26) increased over time, without positive trends in other quality criteria from 1990 through June 2008. Systematic reviews with internal quality evaluation did not meet other quality criteria more often than those that ignored the quality of included studies.

**Conclusion:**

Collaborative efforts from investigators and journal editors are needed to improve the quality of systematic reviews.

## Introduction

Valid epidemiologic research is essential in preventing chronic diseases (1-3). Assessing the quality of observational studies is an important part of evidence synthesis (4). Systematic reviews have become key tools in evidence synthesis from a growing number of epidemiologic studies (5). Producing high-quality systematic reviews is essential to developing generalizable and actionable conclusions (6,7). Quality criteria for systematic reviews have been proposed by working groups that developed the Meta-analysis of Observational Studies in Epidemiology (MOOSE), Strengthening the Reporting of Observational Studies in Epidemiology (STROBE), and a measurement tool for *assessment of multiple systematic reviews* (AMSTAR) (8-12). The working groups and the Cochrane handbook (13) addressed those criteria for systematic reviews that more likely result in biased results, including bias in selection of the studies or the information within studies by the reviewers (14-18) or bias in the publication of positive significant results (6,15,19,20).

Previous research and guidelines (13,21-23) focus on systematic reviews of interventional therapeutic studies. Validity of observational nontherapeutic studies of prevalence of chronic diseases or risk factors for diseases is essential for effective preventive public health actions (24,25). Our aim was to evaluate the quality of systematic reviews of observational nontherapeutic studies that examined the incidence and prevalence of chronic conditions and risk factors for diseases. The criteria we used to determine the reporting and methodologic quality in systematic reviews were from published standards (8-12). We hypothesized that the quality of systematic reviews differs by the time when the study was published, the country in which the study was conducted, the journal of publication, the sponsorship of the study, and whether a conflict of interest was disclosed. We hypothesized also that systematic reviews with internal quality evaluation of the included studies would have better quality, demonstrating commitment to quality of evidence.

## Methods

### Data sources

We searched MEDLINE via PubMed and via Ovid MEDLINE, the Cochrane Library (26) and working groups, WorldCat (27), and Scirus (28) to find systematic reviews of observational nontherapeutic studies published in English from 1966 through June 2008 in core clinical journals (exact search string is listed in Appendix Table 1). We used the definitions of core clinical journals from the *Abridged Index Medicus* (119 indexed titles). We defined observational nontherapeutic studies as observations of patient outcomes that did not examine procedures concerned with the remedial treatment or prevention of diseases (29).

### Study selection

Three investigators independently decided on the eligibility of the studies according to recommendations from the *Cochrane Handbook for Systematic Reviews of Interventions *(13). We reviewed abstracts to exclude comments, expert opinions, letters, case reports, systematic reviews of interventional studies, and systematic reviews of studies of diagnostic accuracy of tests.

### Data extraction

Evaluations of the studies and data extraction were performed independently by 2 researchers. Predefined categorical responses to the checklist items were abstracted into our spreadsheet. Errors in data extraction were assessed by a comparison of the data charts with the original articles (13,30). Any discrepancies were discussed and resolved. The quality criteria that we abstracted were based on guidelines for determining the reporting and methodologic quality of systematic reviews (8-12).

To evaluate selection bias, we abstracted whether the authors of systematic reviews described the search strategy (yes, no, or partially); *yes* indicated that the authors reported time periods of searches, searched databases, and exact search string. We abstracted whether the authors of systematic reviews described study flow (yes, no, or partially); *yes* indicated that the authors reported the list of retrieved citations, the list of excluded studies, and justification for exclusion.

We abstracted as dichotomous variables whether the authors of systematic reviews did any of the following:

- Stated the aim of the review and the primary and secondary hypotheses of the review.
- Included or justified exclusion of articles published in languages other than English.
- Searched for gray literature, including abstracts and unpublished studies, to evaluate publication bias (21).
- Described any contact with authors of the included studies.
- Analyzed sponsorship of and conflict of interest in the included studies.

We abstracted how the authors of systematic reviews described obtained statistical methods with justification and models for pooling with fixed or random effects models in sufficient detail to be replicated (no pooling, random, or fixed). We abstracted whether the authors of pooling analyses reported statistical tests for heterogeneity and whether heterogeneity was statistically significant (not reported, not significant, or significant).

We used 3 categories to classify whether the authors of systematic reviews had evaluated the quality of included studies by using developed or previously published checklists or scales (31): 1) the authors stated planned, formal internal quality evaluations; 2) the authors abstracted selected criteria of external or internal validity without using a planned, formal, and comprehensive internal quality evaluation; and 3) the authors did not conduct internal quality evaluations. We further categorized the studies that evaluated quality criteria to compare studies with no mention of internal quality evaluation of the included studies. We also compared studies with and without planned formal internal quality evaluation. We abstracted with dichotomous responses blinding and reliability testing (reported or not reported) of internal quality evaluations.

We abstracted several explanatory variables that could be related to the quality of systematic reviews:

- The year of publication, defined as a continuous variable. We created categories of 4- or 5-year periods: 1990 to 1994, 1995 to 1999, 2000 to 2004, and 2005 through June 2008.
- The journals of publication.
- The country where the systematic reviews were performed.
- The sponsorship of the reviews. Those that had either governmental or foundational support or were fellowships were defined as having nonprofit support.
- The disclosure of conflict of interest by authors of reviews (either not disclosed, disclosed as no conflict of interest, or disclosed conflict of interest).
- The number of disclosed relationships with industry, defined as a continuous variable.
- The sponsor's participation in data collection, analysis, and interpretation of the results of the review.
- The review outcomes as risk factors for prevalence or incidence of chronic conditions or diseases.

### Data synthesis

We summarized the results in evidence tables. We used prespecified categories of dependent and independent variables and did not force the data into binary categories for definitive tests of significance. We used univariate logistic regression to examine the association between internal quality evaluation and the year of the publication by using the Wald test. Odds ratios (ORs) were calculated with binary logit models and Fisher's scoring method technique. We computed the fractions of systematic reviews meeting various quality criteria in each of the 4 time periods considered. The proportions of systematic reviews that met different levels of each quality criterion were evaluated by using χ^2^ tests and Fisher's exact tests in cases of small numbers. All calculations were performed at 95% confidence intervals (CIs) by using 2-sided *P* values with SAS version 9.1.3 (SAS Institute Inc, Cary, North Carolina).

## Results

We found 145 eligible systematic reviews of observational nontherapeutic studies (study flow in the Appendix Figure) (32-176). The number of published systematic reviews increased from 17 during 1990-1994 to 56 during 2005-2008. Most of the studies were conducted in the United States (55 publications) or in the United Kingdom (28 publications) (Appendix Table 2). Half of the systematic reviews (73 publications) were funded by nonprofit organizations; 56 (39%) reviews did not publish their funding sources, 4 reviews received industry support, and 10 were sponsored jointly by industry and nonprofit organizations. Almost three-fourths (106) of the authors of systematic reviews did not disclose conflict of interest; 35 publications stated that the authors do not have any conflict of interest; and 4 studies were conducted by authors who reported conflict of interest. The studies were published in 49 journals. Most systematic reviews (122 studies) assessed risk factors for chronic diseases, 19 summarized estimates of prevalence or incidence, 2 studies reported prevalence and associations with risk factors, and 2 studies examined levels of risk factors. Most studies reported incidence and risk factors for cardiovascular diseases (46 studies) or cancer (26 studies).

### Quality of systematic reviews

Less than half of the studies reported study flow (49%), assessed gray literature (27%), or addressed language bias (29%) (Table 1). Only 2% of reviews abstracted sponsorship of individual studies and none abstracted the disclosure of conflict of interest by the authors of individual studies that were eligible for the reviews. Pooling was performed in 137 studies; of these, 62% used a random effects model; 57% reported detecting significant heterogeneity across the studies; and 19% did not provide any information about statistical heterogeneity in pooled estimates. The proportion of systematic reviews that met quality criteria including study flow, assessment of gray literature, or the abstraction of funding sources of included studies did not show significant trends from 1990 through 2008. The proportion of systematic reviews that assessed language bias increased from 8% during 1995-1999 to 41% during 2005-2008. In later years, more studies reported using random effects models (79% during 2005-2008 vs 39% during 1995-1999) and tests for statistical heterogeneity (89% during 2005-2008 vs 65% during 1995-1999).

### Internal quality evaluation

Planned and detailed quality assessment of included studies was reported in 37% of systematic reviews, and 18% abstracted more than 1 criterion of external or internal quality; significant positive trends were reported during the evaluated time (Table 1). Quality assessment was masked in 3 studies. Development of the appraisals, including references to previously published tools, was reported in 32 studies, but only 6 tested interobserver agreement for quality assessment.

### Quality of systematic review by explanatory factors

The quality of systematic reviews did not differ much by study location or by the journal of publication. Systematic reviews of prevalence or incidence or risk factors of the diseases did not differ in their quality measures. Sponsorship was not associated with quality of the reviews. The role of conflict of interest was impossible to establish because the authors of 56 reviews did not disclose funding and authors of 106 reviews did not disclose conflict of interest.

### Explanatory factors of internal quality evaluation of included studies

The journal of publication, topic of the review, and continent where the review was conducted were not associated with the likelihood of internal quality evaluation. Systematic reviews of risk factors tended to conduct internal quality evaluation of the included studies more often than reviews of incidence or prevalence or of levels of risk factors. Systematic reviews sponsored by nonprofit organizations conducted internal quality evaluations of individual studies more often than reviews that received corporate funding. Systematic reviews that disclosed conflict of interest conducted internal quality evaluation of individual studies less frequently (10 of 39 studies; 26%) than reviews with no disclosure (44 of 106 studies; 42%). Odds of formal internal quality evaluation (OR, 1.10 per year; 95% CI, 1.02-1.19) and either planned, formal internal quality evaluation or abstraction of quality criteria (OR, 1.17 per year; 95% CI, 1.08-1.26) increased over time. Disclosure of conflict of interest by the authors of systematic reviews was not associated with greater odds of internal quality evaluation.

### Quality of systematic reviews by internal quality evaluation

Complete documentation of the literature search including time period, databases searched, and exact literature search strings was less common among reviews with planned, formal internal quality evaluation (48 studies, 35%) than among reviews without it (90 studies, 65%) (Table 2). However, reviews that either abstracted selected quality criteria or planned, formal internal quality evaluation reported partial (6 studies) or complete (74 studies) information about the literature search more often than studies that did not evaluate quality of included studies (64 studies). Reviews that did not justify exclusion of non-English studies ignored quality of individual studies more often (72 studies) than reviews with planned, formal internal quality evaluation (31 studies). The same pattern was present for publication bias: the reviews that did not mention gray literature also ignored the quality of individual studies. The reviews reporting attempts to contact the authors of included studies either performed planned, formal internal quality evaluation or abstracted selected quality criteria more often than reviews without such attempts (OR, 2.3; 95% CI, 1.1-4.7). Reviews with complete reporting of study flow performed planned, formal internal quality evaluation or abstracted quality criteria more often (51 studies) than reviews without study flows (20 studies). More than half of systematic reviews without planned, formal internal quality evaluation (44 studies) also did not report study flow.

The association between quality of systematic reviews and sponsor participation in the data collection, analyses, and interpretation was difficult to analyze because this information was either omitted or reported in various ways. Less than 10% of systematic reviews contained a clear statement that the sponsors did not play any role in gathering the studies or analyzing or interpreting the results and did not influence the content of the manuscript. Other reviews omitted mention of the role of the sponsor in approval of the manuscript or provided a general statement that sponsors did not influence the conclusions or the content of the paper. Two reviews included statements of unconditional or unrestricted sponsorship of the meta-analyses.

## Discussion

Our analyses showed that less than half of the systematic reviews of nontherapeutic observational studies that were published in core clinical journals met each quality criterion. Quality of systematic reviews did not improve over time. Planned, formal internal quality evaluations of the included studies was reported in less than half of systematic reviews, but the prevalence of internal quality evaluations has increased during the last decade. Our findings are in concordance with previously published methodologic analyses of systematic reviews that also found inconsistent quality and incomplete internal quality evaluation of individual studies (6). Methodologic analyses of systematic reviews that focused on particular diseases or conditions demonstrated that half of the publications had major flaws in design and reporting. For instance, systematic reviews of therapies for renal diseases failed to assess the methodologic quality of included studies (177). Methodologic analyses of systematic reviews of interventions showed that 69% of those randomly selected in MEDLINE meta-analyses did not analyze quality of trials (22). Most (68%) systematic reviews of diagnostic tests for cancer did not provide formal assessments of study quality (178). We also found that the quality of reviews did not differ among types of studies (incidence or risk factors for diseases), types of diseases, or journal of publication.

Journal commitment to high-quality research, however, was associated with improved reporting quality of the publications. For example, adoption by journals of the Consolidated Standards of Reporting Trials (CONSORT) improved the quality of the publications of interventional studies (179,180). An endorsement of the developed standards for observational studies including MOOSE and STROBE checklists may also improve quality of the publications. We did not analyze how many core clinical journals adopted these standards and how quality of the publications changed depending on this adaptation. Peer review of submitted manuscripts should include quality assessment using validated tools (12).

We could not identify the factors that can explain differences in quality of systematic reviews. The role of sponsorship and conflict of interest could not be estimated because of poor reporting of this information. The quality and reliability of quality evaluation of the included studies is unclear because development of the appraisals was described in a small proportion of systematic reviews (32 of 80 studies), and only 6 of 80 studies tested interobserver agreement for quality assessment. We did not evaluate all reviews of observational studies that were published in epidemiologic journals. However, it is unlikely that the quality of reviews published in other journals would be better than those in core clinical journals. Future research should investigate the factors that can explain differences in the quality of systematic reviews.

Peer reviewed publications of high-quality systematic reviews can provide the best available research evidence for evidence-based public health (24). Evidence-based decisions can improve public health practice in preventing incidence and progression of chronic diseases (25). In our analysis, less than half of the systematic reviews of observational nontherapeutic studies met quality criteria established in the MOOSE, STROBE, and AMSTAR statements. Internal quality evaluation of included studies should be an essential part of evidence synthesis, but only half of the reviews reported such evaluation. Collaborative efforts from investigators and journal editors are needed to improve quality of systematic reviews.

## Figures and Tables

**Table 1:** Quality Criteria of Systematic Reviews of Observational Nontherapeutic Studies Published in Core Clinical Journals, by Year of Publication, 1990 Through June 2008

**Evaluated Criteria**	1990-1994, n (N = 17)	1995-1999, n (N = 26)	2000-2004, n (N = 46)	2005-2008, n (N = 56)	Total, n (N = 145)	*P* Value^a^
**Literature search**						
No information	0	0	1	0	1	.7
Documented partially	1	1	3	1	6	
Complete documenting of databases used, exact search strings used, and time periods of searches	16	25	42	55	138	
**Contact with authors of the included studies**						
No information	13	17	31	31	92	.4
The authors of the review attempted to contact the authors of included studies	4	9	15	25	53	
**Study flow**						
Study flow not reported	10	15	29	18	72	.04
Study flow partially reported	0	0	0	2	2	
Study flow reported with the list of retrieved citations, the list of excluded studies, and justification for exclusion for each study	7	11	17	36	71	
**Articles published in languages other than English**						
Language bias was not addressed	15	24	31	33	103	.01
Language bias was addressed: the authors included or justified exclusion of the non-English publications	2	2	15	23	42	
**Gray literature**						
Gray literature was not assessed	15	17	36	38	106	.25
Reporting of the method of handling abstracts and unpublished studies	2	9	10	18	39	
**Conflict of interest from included studies**						
Conflict of interest in included studies was not abstracted	17	26	46	56	145	NA
**Sponsorship of the included studies**						
Sponsorship of included studies was not analyzed	16	25	46	55	142	.45
Sponsorship of included studies was analyzed	1	1	0	1	3	
**Pooled model obtained in the review**						
Pooling was not obtained	2	0	4	2	8	<.001
Fixed effects model was obtained for meta-analyses	10	16	11	10	47	
Random effects model was obtained for meta-analyses	5	10	31	44	90	
**Heterogeneity across included studies**						
Heterogeneity across studies was not reported	6	9	7	6	28	.04
Heterogeneity across studies was not significant	5	6	13	11	35	
Heterogeneity across studies was significant	6	11	26	39	82	
**Formal internal quality evaluation of included studies**						
Planned, formal internal quality evaluation with developed or previously published checklists or scales	3	6	20	25	54	<.001
Some selected criteria of external or internal quality of included studies were abstracted without planned, formal internal quality evaluation	2	3	1	20	26	
No internal quality evaluation	12	17	25	11	65	
**Reliability of internal quality evaluation reported**	2	4	8	18	32	.99
**Internal quality evaluation was masked**	1	1	0	1	3	.11

Abbreviation: NA, not applicable.

a *P* values for overall χ^2^ test.

**Table 2:** Quality of Systematic Reviews, by Internal Quality Evaluation of Included Studies, 1990 Through June 2008

Quality Criterion	Definition of Formal Internal Quality Evaluation			
	Planned, Formal Internal Quality Evaluation or Abstraction of Some Quality Criteria, n	Neither Planned, Formal Internal Quality Evaluation nor Abstraction of Some Quality Criteria, n	Planned, Formal Internal Quality Evaluation, n	No Planned, Formal Internal Quality Evaluation, n
**Literature search**	*P* = .04^a^		*P* = .004^b^	
No information	0	1	0	1
Documented partially	6	0	6	0
Complete documenting of databases used, exact search strings used, and time periods of searches	74	64	48	90
**Contact with authors of the included studies**	*P* = .02^a^		*P* = .25^b^	
No information	44	48	31	61
The authors of the review attempted to contact the authors of included studies	36	17	23	30
**Study flow**	*P* < .001^a^		*P* = .003^b^	
Study flow not reported	28	44	17	55
Study flow partially reported	1	1	1	1
Study flow reported with the list of retrieved citations, the list of excluded studies, and justification for exclusion for each study	51	20	36	35
**Articles published in languages other than English**	*P* = .001^a^		*P* = .01^b^	
No information	48	55	31	72
Inclusion of non-English studies or justification for exclusion	32	10	23	19
**Gray literature**	*P* = .09^a^		*P* = .04^b^	
No information	54	52	34	72
Reporting of the method of handling abstracts and unpublished studies	26	13	20	19
**Conflict of interest from included studies**				
No information	80	65	54	91
**Sponsorship of the included studies**	*P* = .44^a^		*P* = .18^b^	
No information	79	63	54	88
Sponsorship of included studies was abstracted	1	2	0	3
**Pooled model obtained in the review**	*P* < .001^a^		*P* = .06^b^	
Not applicable (no pooling)	6	2	5	3
Fixed effects model	15	32	12	35
Random effects model	59	31	37	53
**Heterogeneity across included studies**	*P* = .27^a^		*P* = .67^b^	
Not reported	13	15	9	19
Heterogeneity was not significant	17	18	15	20
Heterogeneity was significant at least for one association	50	32	30	52

a *P* value for overall χ^2^ test between planned, formal internal quality evaluation or abstraction of some quality criteria versus neither planned, formal internal quality evaluation nor abstraction of some quality criteria.

b *P* value for overall χ^2^ test between planned, formal internal quality evaluation versus no planned, formal internal quality evaluation.

**Figure 1:**
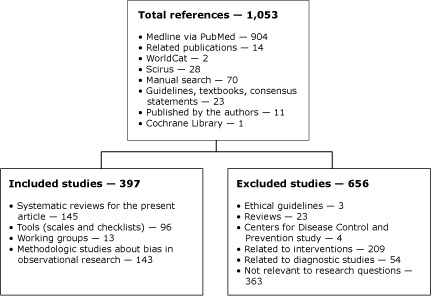
Study flow to identify systematic reviews of observational studies, scales, and checklists for planned formal internal quality evaluation, and studies about bias in observational research, 1990 through June 2008.

**Table:** 

This flow chart shows 3 boxes. The top box reads
**Total references — 1,053**
Medline via PubMed — 904 Related publications — 14 WorldCat — 2 Scirus — 28 Manual search — 70 Guidelines, textbooks, consensus statements — 23 Published by the authors — 11 Cochrane Library — 1
It is connected by a vertical line to 2 additional boxes. The left box reads
**Included studies — 397**
Systematic reviews for the present article — 145 Tools (scales and checklists) — 96 Working groups — 13 Methodologic studies about bias in observational research — 143
The right box reads
**Excluded studies — 656**
Ethical guidelines — 3 Reviews — 23 Centers for Disease Control and Prevention study — 4 Related to interventions — 209 Related to diagnostic studies — 54 Not relevant to research questions — 363

## Appendix

**Table 1:** Search Strategy and Exact Search Strings Used to Identify Systematic Reviews of Observational Studies, Scales and Checklists for Internal Quality Evaluation, and Studies About Bias in Observational Research, 1966 Through June 2008

Search Method	No. of Articles Identified
**Search strategy for Ovid MEDLINE**	
1. exp Research Design/st [Standards]	4,303
2. exp Chronic Disease/ep [Epidemiology]	1,619
3. exp Urinary Incontinence/ep [Epidemiology]	1,155
4. exp Fecal Incontinence/ep [Epidemiology]	328
5. exp "Sleep Initiation and Maintenance Disorders"/ep [Epidemiology]	565
6. exp Depression/ep [Epidemiology]	4,700
7. exp Depressive Disorder/ep [Epidemiology]	6,816
8. exp Myocardial Infarction/	43,531
9. 6 or 7	11,214
10. 8 and 9	105
11. 2 or 3 or 4 or 5 or 10	3,636
12. 1 and 11	9
13. exp Data Collection/mt, st [Methods, Standards]	36,173
14. exp "Bias (Epidemiology)"/	25,369
15. exp Questionnaires/st [Standards]	3,879
16. exp Evidence-Based Medicine/	27,487
17. 13 or 14 or 15 or 16	86,857
18. 11 and 17	127
19. 12 or 18	133
20. limit 19 to english language	124
21. exp "Predictive Value of Tests"/	62,290
22. exp "Reproducibility of Results"/	126,475
23. 21 or 22	182,941
24. 11 and 23	126
25. limit 24 to english language	121
26. 20 or 25	224
27. exp randomized controlled trial/	151,027
28. 11 and 27	74
29. exp research design/	134,468
30. 28 and 29	15
31. 1 and 16	547
32. ep.fs.	434,923
33. exp epidemiology/	6,500
34. 32 or 33	437,784
35. 31 and 34	29
36. exp incidence/	81,260
37. exp prevalence/	83,713
38. 36 or 37	157,239
39. 31 and 38	14
40. 26 or 30 or 35 or 39	268
41. limit 40 to english language	267
42. limit 41 to journal article	251
43. from 42 keep 1-251	251
**MEDLINE search via PubMed**	
("Biomedical Research/methods"[MeSH] OR "Biomedical Research/organization and administration"[MeSH] OR "Biomedical Research/standards"[MeSH] OR "Biomedical Research/statistics and numerical data"[MeSH] OR "Biomedical Research/trends"[MeSH]) Limits: Humans, Journal Article, English	3,703
"Epidemiologic Studies"[MeSH] AND "Research Design/standards"[MeSH] AND ("Evaluation Studies as Topic/classification"[MeSH] OR "Evaluation Studies as Topic/methods"[MeSH] OR "Evaluation Studies as Topic/standards"[MeSH]) Limits: Humans, Journal Article, English	59
"Publishing/standards"[MeSH] AND "Epidemiologic Methods"[MeSH] AND "Research Design/standards"[MeSH] Limits: Humans, Journal Article, English	65
"STROBE Initiative"[Corporate Author]	10
"Bias (Epidemiology)"[MeSH] AND "Epidemiologic Studies"[MeSH] AND "Epidemiologic Methods"[MeSH] AND "Research Design/standards"[MeSH] Limits: Humans, Journal Article, English	97
"Evidence-Based Medicine"[MeSH] AND "Epidemiologic Studies"[MeSH] AND "Epidemiologic Methods"[MeSH] AND "Research Design/standards"[MeSH] Limits: Humans, Journal Article, English	25
"Research Design/standards"[MeSH] AND "Epidemiologic Studies"[MeSH] AND "Epidemiologic Measurements"[MeSH] AND "Bias (Epidemiology)"[MeSH] Limits: Humans, Journal Article, English AND "Incidence"[MeSH] Limits: Humans, Journal Article, English	8
"Research Design/standards"[MeSH] AND "Epidemiologic Studies"[MeSH] AND "Epidemiologic Measurements"[MeSH] AND "Bias (Epidemiology)"[MeSH] Limits: Humans, Journal Article, English AND "Prevalence"[MeSH] Limits: Humans, Journal Article, English	7
("Prevalence"[MeSH]) AND systematic[sb] "Working group" Limits: English	15
[CN] Limits: Humans, Meta-Analysis, English, Core clinical journals	2
("Prevalence"[MeSH]) AND systematic[sb] Limits: Humans, Meta-Analysis, English, Core clinical journals	83
Moher D[author]	198
"Epidemiologic Studies"[MeSH] Limits: Humans, Meta-Analysis, English AND "Incidence"[MeSH] Limits: Humans, Meta-Analysis, English Limits: Humans, Meta-Analysis, English, Core clinical journals	57
"Epidemiologic Studies"[MeSH] AND "Incidence"[MeSH] Limits: Humans, Meta-Analysis, English	236
"Epidemiologic Studies"[MeSH] AND "Incidence"[MeSH] AND Evidence Limits: Humans, Meta-Analysis, English	52
"Incidence"[MeSH] Limits: Humans, Meta-Analysis, English	635
"Risk"[MeSH] AND "Epidemiologic Studies"[MeSH] Limits: Humans, Meta-Analysis, English, Core clinical journals	273
"Prevalence"[MeSH] Limits: Humans, Meta-Analysis, English, Core clinical journals	84
Altman DG[author]	7
Higgins J[author]	3
"Review Literature as Topic"[MeSH] AND "Research Design/standards"[MeSH] AND "Epidemiologic Studies"[MeSH] Limits: Humans, English, Core clinical journals	0
"Review Literature as Topic"[MeSH] AND "Epidemiologic Studies"[MeSH] AND "Quality control"[MeSH]	1
"Incidence"[MeSH] AND "Chronic Disease/epidemiology"[MeSH] AND "Peer Review, Research"[MeSH] AND "Research Design/standards"[MeSH]	0
"Incidence"[MeSH] AND "Chronic Disease/epidemiology"[MeSH] AND "Peer Review, Research"[MeSH]	0
"Incidence"[MeSH] AND "Chronic Disease/epidemiology"[MeSH] AND "Research Design/standards"[MeSH]	0
"Incidence"[MeSH] AND "Chronic Disease/epidemiology"[MeSH] AND ("Data Collection/methods"[MeSH] OR "Data Collection/standards"[MeSH])	5
"Incidence"[MeSH] AND "Chronic Disease/epidemiology"[MeSH] AND "Bias (Epidemiology)"[MeSH]	1
"Incidence"[MeSH] AND "Chronic Disease/epidemiology"[MeSH] AND ("Questionnaires/methods"[MeSH] OR "Questionnaires/standards"[MeSH])	0
"Incidence"[MeSH] AND "Chronic Disease/epidemiology"[MeSH] AND "Evidence-Based Medicine"[MeSH]	2
"Incidence"[MeSH] AND "Chronic Disease/epidemiology"[MeSH] AND "Reproducibility of Results"[MeSH]	3
"Prevalence"[MeSH] AND "Chronic Disease/epidemiology"[MeSH] AND "Peer Review, Research"[MeSH] AND "Research Design/standards"[MeSH]	0
"Prevalence"[MeSH] AND "Chronic Disease/epidemiology"[MeSH] AND "Peer Review, Research"[MeSH]	0
"Prevalence"[MeSH] AND "Chronic Disease/epidemiology"[MeSH] AND "Research Design/standards"[MeSH]	0
"Prevalence"[MeSH] AND "Chronic Disease/epidemiology"[MeSH] AND ("Data Collection/methods"[MeSH] OR "Data Collection/standards"[MeSH])	16
"Prevalence"[MeSH] AND "Chronic Disease/epidemiology"[MeSH] AND "Bias (Epidemiology)"[MeSH]	6
"Prevalence"[MeSH] AND "Chronic Disease/epidemiology"[MeSH] AND ("Questionnaires/methods"[MeSH] OR "Questionnaires/standards"[MeSH])	1
"Prevalence"[MeSH] AND "Chronic Disease/epidemiology"[MeSH] AND "Evidence-Based Medicine"[MeSH]	0
"Prevalence"[MeSH] AND "Chronic Disease/epidemiology"[MeSH] AND "Reproducibility of Results"[MeSH]	12
"Risk Factors"[MeSH] AND "Chronic Disease/epidemiology"[MeSH] AND "Peer Review, Research"[MeSH] AND "Research Design/standards"[MeSH]	0
"Risk Factors"[MeSH] AND "Chronic Disease/epidemiology"[MeSH] AND "Peer Review, Research"[MeSH]	0
"Risk Factors"[MeSH] AND "Chronic Disease/epidemiology"[MeSH] AND "Research Design/standards"[MeSH]	1
"Risk Factors"[MeSH] AND "Chronic Disease/epidemiology"[MeSH] AND ("Data Collection/methods"[MeSH] OR "Data Collection/standards"[MeSH])	18
"Risk Factors"[MeSH] AND "Chronic Disease/epidemiology"[MeSH] AND "Bias (Epidemiology)"[MeSH]	7
"Risk Factors"[MeSH] AND "Chronic Disease/epidemiology"[MeSH] AND ("Questionnaires/methods"[MeSH] OR "Questionnaires/standards"[MeSH])	1
"Risk Factors"[MeSH] AND "Chronic Disease/epidemiology"[MeSH] AND "Evidence-Based Medicine"[MeSH]	4
"Risk Factors"[MeSH] AND "Chronic Disease/epidemiology"[MeSH] AND "Reproducibility of Results"[MeSH]	10
"Health Care Quality, Access, and Evaluation"[MeSH] AND "Chronic Disease/epidemiology"[MeSH] AND "Peer Review, Research"[MeSH] AND "Research Design/standards"[MeSH]	0
"Health Care Quality, Access, and Evaluation"[MeSH] AND "Chronic Disease/epidemiology"[MeSH] AND "Peer Review, Research"[MeSH]	0
"Health Care Quality, Access, and Evaluation"[MeSH] AND "Chronic Disease/epidemiology"[MeSH] AND "Research Design/standards"[MeSH]	4
"Health Care Quality, Access, and Evaluation"[MeSH] AND "Chronic Disease/epidemiology"[MeSH] AND "Evidence-Based Medicine"[MeSH]	8
"Health Care Quality, Access, and Evaluation"[MeSH] AND "Chronic Disease/epidemiology"[MeSH] AND "Bias (Epidemiology)"[MeSH]	33
"Models, Statistical"[MeSH] AND "Risk Factors"[MeSH] AND "Chronic Disease/epidemiology"[MeSH] AND "Research Design/standards"[MeSH]	0
"Models, Statistical"[MeSH] AND "Incidence"[MeSH] AND "Chronic Disease/epidemiology"[MeSH] AND "Research Design/standards"[MeSH]	0
"Models, Statistical"[MeSH] AND "Prevalence"[MeSH] AND "Chronic Disease/epidemiology"[MeSH] AND "Research Design/standards"[MeSH]	0
"Epidemiologic Studies"[MeSH] AND "Models, Statistical"[MeSH] AND "Research Design/standards"[MeSH]	47
"Prevalence"[MeSH] AND "Epidemiologic Studies"[MeSH] AND "Models, Statistical"[MeSH] AND "Bias (Epidemiology)"[MeSH]	61
"Incidence"[MeSH] AND "Epidemiologic Studies"[MeSH] AND "Models, Statistical"[MeSH] AND "Bias (Epidemiology)"[MeSH]	66
"Research Design/standards"[MeSH] AND ("Biomedical Research/methods"[MeSH] OR "Biomedical Research/organization and administration"[MeSH] OR "Biomedical Research/standards"[MeSH] OR "Biomedical Research/statistics and numerical data"[MeSH] OR "Biomedical Research/trends"[MeSH]) Limits: Humans, Journal Article, English	62

Abbreviations: MeSH, Medical Subject Heading term; sb, subset; CN, corporate author.

**Table 2:** Quality of Systematic Review and Meta-Analyses of Nontherapeutic Observational Studies Published in Core Clinical Journals, 1990 through June 2008

**Publication Characteristics**	Outcome	Estimate	Assessment of Quality of Included Studies
**Bracken, 1990 (32)** **Country:** United States **Journal:** Obstet Gynecol **Sponsorship:** Not reported **Conflict of interest (COI):** Not reported **Sponsor participation in data analyses:** Not reported	Congenital malformations in offspring	Risk	No
**Romieu et al, 1990 (33)** **Country:** United States **Journal:** Cancer **Sponsorship:** Government **COI:** Not reported **Sponsor participation in data analyses:** Not reported	Breast cancer	Risk	Quality criteria abstracted
**Haughey et al, 1992 (34)** **Country:** United States **Journal:** Ann Otol Rhinol Laryngol **Sponsorship:** Not reported **COI:** Not reported **Sponsor participation in data analyses:** Not reported	Second malignant tumors in head and neck cancer	Risk	No
**Lemon et al, 1992 (35)** **Country:** United States **Journal:** Cancer **Sponsorship:** Government **COI:** Not reported **Sponsor participation in data analyses:** Not reported	Nonfamilial breast cancer	Continuous variable	No
**McKenna, 1992 (36)** **Country:** United Kingdom **Journal:** Am J Med **Sponsorship:** Nonprofit organization, nursing home **COI:** Not reported **Sponsor participation in data analyses:** Not reported	Differences in vitamin D status	Prevalence	Quality criteria abstracted
**Morris et al, 1992 (37)** **Country:** United States **Journal:** Am J Public Health **Sponsorship:** Not reported **COI:** Not reported **Sponsor participation in data analyses:** Not reported	Cancer	Risk	Yes
**Myers and Basinski, 1992 (38)** **Country: Canada** **Journal:** Arch Intern Med **Sponsorship:** Nonprofit organization, award **COI:** Not reported **Sponsor participation in data analyses:** Not reported	Coronary heart disease	Risk	No
**Becker et al, 1993 (39)** **Country:** United States **Journal:** Ann Emerg Med **Sponsorship:** Not reported **COI:** Not reported **Sponsor participation in data analyses:** Not reported	Survival of cardiac arrest	Risk	No
**Brownson et al, 1993 (40)** **Country:** United States **Journal:** Arch Intern Med **Sponsorship:** Not reported **COI:** Not reported **Sponsor participation in data analyses:** Not reported	Adult leukemia	Risk	Yes
**Ernst and Resch, 1993 (41)** **Country:** Austria **Journal:** Ann Intern Med **Sponsorship:** Not reported **COI:** Not reported **Sponsor participation in data analyses:** Not reported	Cardiovascular risk factor	Risk	No
**Katerndahl, 1993 (42)** **Country:** United States **Journal:** J Nerv Ment Dis **Sponsorship:** Not reported **COI:** Not reported **Sponsor participation in data analyses:** Not reported	Panic disorder and mitral valve prolapse	Risk	Yes
**Harris and Barraclough, 1994 (43)** **Country:** United Kingdom **Journal:** Medicine **Sponsorship:** Industry **COI:** Not reported **Sponsor participation in data analyses:** Not reported	Suicide	Risk	No
**Kawachi et al, 1994 (44)** **Country:** United States **Journal:** Br Heart J **Sponsorship:** Industry, scholarship **COI:** Not reported **Sponsor participation in data analyses:** Not reported	Coronary heart disease	Risk	No
**Law et al, 1994 (45)** **Country:** United Kingdom **Journal:** BMJ **Sponsorship:** Not reported **COI:** Not reported **Sponsor participation in data analyses:** Not reported	Hazards of reducing serum cholesterol	Risk	No
**Law et al, 1994 (46)** **Country:** United Kingdom **Journal:** BMJ **Sponsorship:** Not reported **COI:** Not reported **Sponsor participation in data analyses:** Not reported	Ischemic heart disease	Risk	No
**Steffen et al, 1994 (47)** **Country:** Switzerland **Journal:** JAMA **Sponsorship:** Not reported **COI:** Not reported **Sponsor participation in data analyses:** Not reported	Hepatitis A	Risk	No
**Zhang and Begg, 1994 (48)** **Country:** United States **Journal:** Int J Epidemiol **Sponsorship:** Government **COI:** Not reported **Sponsor participation in data analyses:** Not reported	Cervical neoplasia	Risk	No
**Everhart and Wright, 1995 (49)** **Country:** United States **Journal:** JAMA **Sponsorship:** Not reported **COI:** Not reported **Sponsor participation in data analyses:** Not reported	Pancreatic cancer	Risk	No
**Feinberg et al, 1995 (50)** **Country:** United States **Journal:** Arch Intern Med **Sponsorship:** Not reported **COI:** Not reported **Sponsor participation in data analyses:** Not reported	Atrial fibrillation	Prevalence	No
**Ritchie and Kildea, 1995 (51)** **Country:** France **Journal:** Lancet **Sponsorship:** Not reported **COI:** Not reported **Sponsor participation in data analyses:** Not reported	Senile dementia	Prevalence	No
**Raman-Wilms et al, 1995 (52)** **Country:** Canada **Journal:** Obstet Gynecol **Sponsorship:** Not reported **COI:** Not reported **Sponsor participation in data analyses:** Not reported	Fetal genital effects	Risk	No
**Hatsukami and Fischman, 1996 (53)** **Country:** United States **Journal:** JAMA **Sponsorship:** Not reported **COI:** Not reported **Sponsor participation in data analyses:** Not reported	Use of crack cocaine and cocaine hydrochloride	Prevalence	No
**Hill and Schoener, 1996 (54)** **Country:** United States **Journal:** Am J Psychiatry **Sponsorship:** Not reported **COI:** Not reported **Sponsor participation in data analyses:** Not reported	Attention deficit hyperactivity disorder	Prevalence	No
**Hackshaw et al, 1997 (55)** **Country:** United Kingdom **Journal:** BMJ **Sponsorship:** Government **COI:** Reported as not a conflict of interest **Sponsor participation in data analyses:** "The views expressed are those of the authors and not necessarily those of the Department of Health."	Lung cancer	Risk	No
**Kluijtmans et al, 1997 (56)** **Country:** Netherlands **Journal:** Circulation **Sponsorship:** Nonprofit organization, industry **COI:** Not reported **Sponsor participation in data analyses:** Not reported	Coronary artery disease	Risk	No
**Law and Hackshaw, 1997 (57)** **Country:** United Kingdom **Journal:** BMJ **Sponsorship:** None **COI:** Reported as not a conflict of interest **Sponsor participation in data analyses:** None	Hip fracture	Risk	No
**Law et al, 1997 (58)** **Country:** United Kingdom **Journal:** BMJ **Sponsorship:** Government **COI:** Reported as not a conflict of interest **Sponsor participation in data analyses:** "The Department of Health (England) supported this work, although the views are our own."	Ischemic heart disease	Risk	No
**Danesh et al, 1998 (59)** **Country:** United Kingdom **Journal:** JAMA **Sponsorship:** Scholarship, nonprofit organization **COI:** Not reported **Sponsor participation in data analyses:** Not reported	Coronary heart disease	Risk	Yes
**French and Brocklehurst, 1998 (60)** **Country:** United Kingdom **Journal:** Br J Obstet Gynaecol **Sponsorship:** Not reported **COI:** Not reported **Sponsor participation in data analyses:** Not reported	Survival in women infected with human immunodeficiency virus	Risk	Yes
**Forgie et al, 1998 (61)** **Country:** Canada **Journal:** Arch Intern Med **Sponsorship:** Industry, government, fellowships, nonprofit organization **COI:** Not reported **Sponsor participation in data analyses:** Not reported	Allogeneic blood transfusion	Risk	No
**Huang et al, 1998 (62)** **Country:** Canada **Journal:** Gastroenterology **Sponsorship:** Not reported **COI:** Not reported **Sponsor participation in data analyses:** Not reported	Gastric cancer	Risk	Yes
**Johnston et al, 1998 (63)** **Country:** United States **Journal:** Neurology **Sponsorship:** Not reported **COI:** Not reported **Sponsor participation in data analyses:** Not reported	Subarachnoid hemorrhage	Risk	No
**Lazarou et al, 1998 (64)** **Country:** Canada **Journal:** JAMA **Sponsorship:** Scholarship, nonprofit organization **COI:** Not reported **Sponsor participation in data analyses:** Not reported	Adverse drug reactions in hospitalized patients	Prevalence	Quality criteria abstracted
**Ray, 1998 (65)** **Country:** Canada **Journal:** Arch Intern Med **Sponsorship:** Not reported **COI:** Not reported **Sponsor participation in data analyses:** Not reported	Venous thromboembolic disease	Risk	Quality criteria abstracted
**Spencer-Green, 1998 (66)** **Country:** United States **Journal:** Arch Intern Med **Sponsorship:** Not reported **COI:** Not reported **Sponsor participation in data analyses:** Not reported	Secondary diseases from primary Reynaud phenomenon	Risk	Yes
**Stratton et al, 1998 (67)** **Country:** United Kingdom **Journal:** Br J Obstet Gynaecol **Sponsorship:** Research fellowship, nonprofit organization **COI:** Not reported **Sponsor participation in data analyses:** Not reported	Ovarian cancer	Risk	No
**Zock and Katan, 1998 (68)** **Country:** Netherlands **Journal:** Am J Clin Nutr **Sponsorship:** Nonprofit organization **COI:** Not reported **Sponsor participation in data analyses:** Not reported	Breast, colorectal, and prostate cancer	Risk	Quality criteria abstracted
**Zondervan et al, 1998 (69)** **Country:** United Kingdom **Journal:** Br J Obstet Gynaecol **Sponsorship:** Nonprofit organization **COI:** Not reported **Sponsor participation in data analyses:** Not reported	Chronic pelvic pain in women	Prevalence	No
**Angelillo and Villari, 1999 (70)** **Country:** Italy **Journal:** Bull World Health Organ **Sponsorship:** Not reported **COI:** Not reported **Sponsor participation in data analyses:** Not reported	Childhood leukemia	Risk	Yes
**He et al, 1999 (71)** **Country:** United States **Journal:** N Engl J Med **Sponsorship:** Nonprofit organization **COI:** Not reported **Sponsor participation in data analyses:** Not reported	Coronary heart disease	Risk	No
**Shaffer et al, 1999 (72)** **Country:** United States **Journal:** Am J Public Health **Sponsorship:** Nonprofit organization **COI:** Not reported **Sponsor participation in data analyses:** Not reported	Disordered gambling behavior	Prevalence	No
**Wittrup et al, 1999 (73)** **Country:** Denmark **Journal:** Circulation **Sponsorship:** Government **COI:** Not reported **Sponsor participation in data analyses:** Not reported	Ischemic heart disease	Risk	Yes
**Yoder et al, 1999 (74)** **Country:** United States **Journal:** Obstet Gynecol **Sponsorship:** Not reported **COI:** Not reported **Sponsor participation in data analyses:** Not reported	Fetus with isolated choroid plexus cysts	Risk	No
**Christen et al, 2000 (75)** **Country:** United States **Journal:** Arch Intern Med **Sponsorship:** Not reported **COI:** Not reported **Sponsor participation in data analyses:** Not reported	Cardiovascular disease	Risk	Yes
**Cleophas et al, 2000 (76)** **Country:** Netherlands **Journal:** Am J Cardiol **Sponsorship:** Not reported **COI:** Not reported **Sponsor participation in data analyses:** Not reported	Coronary artery disease	Risk	Yes
**DiMatteo et al, 2000 (77)** **Country:** United States **Journal:** Arch Intern Med **Sponsorship:** Industry, scholarship **COI:** Not reported **Sponsor participation in data analyses:** Not reported	Noncompliance with medical treatment	Risk	Quality criteria abstracted
**WHO Collaborative Study Team on the Role of Breastfeeding on the Prevention of Infant Mortality, 2000 (78)** **Country:** Brazil **Journal:** Lancet **Sponsorship:** Nonprofit organization **COI:** Not reported **Sponsor participation in data analyses:** Not reported	Infant and child mortality	Risk	No
**Wilson et al, 2000 (79)** **Country:** Canada **Journal:** Arch Intern Med **Sponsorship:** Not reported **COI:** Not reported **Sponsor participation in data analyses:** Not reported	Mortality after myocardial infarction	Risk	Yes
**Zeegers et al, 2000 (80)** **Country:** Netherlands **Journal:** Cancer **Sponsorship:** Not reported **COI:** Not reported **Sponsor participation in data analyses:** Not reported	Urinary tract cancer	Risk	Yes
**Danesh et al, 2001 (81)** **Country:** United Kingdom **Journal:** Circulation **Sponsorship:** Government, scholarship **COI:** Not reported **Sponsor participation in data analyses:** Not reported	Coronary heart disease	Risk	No
**Eaden et al, 2001 (82)** **Country:** United Kingdom **Journal:** Gut **Sponsorship:** Nonprofit organization **COI:** Not reported **Sponsor participation in data analyses:** Not reported	Colorectal cancer	Risk	Yes
**Faraone et al, 2001 (83)** **Country:** United States **Journal:** Am J Psychiatry **Sponsorship:** Government **COI:** Not reported **Sponsor participation in data analyses:** Not reported	Attention deficit hyperactivity disorder	Risk	Yes
**Horta et al, 2001 (84)** **Country:** Brazil **Journal:** Am J Public Health **Sponsorship:** Government **COI:** Not reported **Sponsor participation in data analyses:** Not reported	Early weaning	Risk	Yes
**Rebora, 2001 (85)** **Country:** Italy **Journal:** Arch Dermatol **Sponsorship:** Not reported **COI:** Not reported **Sponsor participation in data analyses:** Not reported	Coronary artery disease	Risk	Yes
**Cannon et al, 2002 (86)** **Country:** United Kingdom **Journal:** Am J Psychiatry **Sponsorship:** Research fellowship, nonprofit organization **COI:** Not reported **Sponsor participation in data analyses:** Not reported	Schizophrenia	Risk	No
**Hellermann et al, 2002 (87)** **Country:** United States **Journal:** Am J Med **Sponsorship:** Government, nonprofit organization, fellowship **COI:** Not reported **Sponsor participation in data analyses:** Not reported	Heart failure	Risk	No
**Huang et al, 2002 (88)** **Country:** Canada **Journal:** Lancet **Sponsorship:** Not reported **COI:** Reported as not a conflict of interest **Sponsor participation in data analyses:** Not reported	Peptic-ulcer disease	Risk	Yes
**Huncharek et al, 2002 (89)** **Country:** United States **Journal:** Am J Public Health **Sponsorship:** Nonprofit organization, industry **COI:** Not reported **Sponsor participation in data analyses:** Not reported	Malignant melanoma	Risk	Yes
**Juul et al, 2002 (90)** **Country:** Denmark **Journal:** Blood **Sponsorship:** Government, nonprofit organization **COI:** Not reported **Sponsor participation in data analyses:** "They had no role in gathering, analyzing, or interpreting the data and had no right to approve or disapprove the submitted paper."	Factor V Leiden	Risk	Yes
**Kelly et al, 2002 (91)** **Country:** United States **Journal:** Neurology **Sponsorship:** Nonprofit organization, industry, fellowship **COI:** Not reported **Sponsor participation in data analyses:** Not reported	Risk of ischemic stroke	Risk	No
**Klerk et al, 2002 (92)** **Country:** Netherlands **Journal:** JAMA **Sponsorship:** Government, "public/private partnership" **COI:** Not reported **Sponsor participation in data analyses:** Not reported	Coronary heart disease	Risk	Yes
**Kozer et al, 2002 (93)** **Country:** Canada **Journal:** Am J Obstet Gynecol **Sponsorship:** Industry **COI:** Not reported **Sponsor participation in data analyses:** Not reported	Congenital anomalies	Risk	No
**Law et al, 2002 (94)** **Country:** United Kingdom **Journal:** Arch Intern Med **Sponsorship:** Not reported **COI:** Not reported **Sponsor participation in data analyses:** Not reported	Death after myocardial infarction	Risk	No
**Wald et al, 2002 (95)** **Country:** United Kingdom **Journal:** BMJ **Sponsorship:** None **COI:** Reported as not a conflict of interest **Sponsor participation in data analyses:** None	Cardiovascular disease	Risk	No
**Wald and Link, 2002 (96)** **Country:** United States **Journal:** J Infect Dis **Sponsorship:** Government **COI:** Not reported **Sponsor participation in data analyses:** Not reported	Human immunodeficiency virus infection	Risk	No
**Benjamin et al, 2003 (97)** **Country:** United States **Journal:** Pediatrics **Sponsorship:** Government **COI:** Not reported **Sponsor participation in data analyses:** Not reported	End-organ damage	Prevalence	No
**Clarfield, 2003 (98)** **Country:** Israel **Journal:** Arch Intern Med **Sponsorship:** Not reported **COI:** Reported as not a conflict of interest **Sponsor participation in data analyses:** Not reported	Reversible dementias	Prevalence	No
**Cole and Dendukuri, 2003 (99)** **Country:** Canada **Journal:** Am J Psychiatry **Sponsorship:** Not reported **COI:** Not reported **Sponsor participation in data analyses:** Not reported	Depression among elderly community subjects	Risk	Yes
**Gisbert et al, 2003 (100)** **Country:** Spain **Journal:** Gastroenterology **Sponsorship:** Nonprofit organization **COI:** Not reported **Sponsor participation in data analyses:** Not reported	Hepatitis C virus infection	Risk	Yes
**Glatt et al, 2003 (101)** **Country:** United States **Journal:** Am J Psychiatry **Sponsorship:** Government **COI:** Not reported **Sponsor participation in data analyses:** Not reported	Schizophrenia	Risk	No
**Halbert et al, 2003 (102)** **Country:** United States **Journal:** Chest **Sponsorship:** Industry **COI:** Not reported **Sponsor participation in data analyses:** Not reported	Prevalence estimates for chronic obstructive pulmonary disease	Prevalence	No
**Huang et al, 2003 (103)** **Country:** Canada **Journal:** Gastroenterology **Sponsorship:** Not reported **COI:** Not reported **Sponsor participation in data analyses:** Not reported	Gastric cancer	Risk	Yes
**Rey et al, 2003 (104)** **Country:** Canada **Journal:** Lancet **Sponsorship:** Government **COI:** Reported as not a conflict of interest **Sponsor participation in data analyses:** Not reported	Fetal loss	Risk	Yes
**Riboli and Norat, 2003 (105)** **Country:** France **Journal:** Am J Clin Nutr **Sponsorship:** Government **COI:** Reported as not a conflict of interest **Sponsor participation in data analyses:** Not reported	Cancer risk	Risk	No
**Scholten-Peeters et al, 2003 (106)** **Country:** Netherlands **Journal:** Pain **Sponsorship:** Not reported **COI:** Not reported **Sponsor participation in data analyses:** Not reported	Whiplash-associated disorders	Risk	Yes
**Thurnham et al, 2003 (107)** **Country:** United Kingdom **Journal:** Lancet **Sponsorship:** Government, fellowship **COI:** Reported as not a conflict of interest **Sponsor participation in data analyses:** "The funding source had no role in study design, data collection, data analysis, data interpretation, or in the writing of this report."	Vitamin A deficiency	Continuous variable	No
**Zeegers et al, 2003 (108)** **Country:** Netherlands **Journal:** Cancer **Sponsorship:** Government **COI:** Not reported **Sponsor participation in data analyses:** Not reported	Prostate carcinoma	Risk	No
**Burzotta et al, 2004 (109)** **Country:** Italy **Journal:** Heart **Sponsorship:** Fellowship **COI:** Not reported **Sponsor participation in data analyses:** Not reported	Coronary ischemic syndromes	Risk	No
**Casas et al, 2004 (110)** **Country:** United Kingdom **Journal:** Circulation **Sponsorship:** Government, 1 author holds a chair of nonprofit organization **COI:** Not reported **Sponsor participation in data analyses:** Not reported	Ischemic heart disease	Risk	No
**Casas et al, 2004 (111)** **Country:** United Kingdom **Journal:** Arch Neurol **Sponsorship:** Fellowship **COI:** Not reported **Sponsor participation in data analyses:** Not reported	Ischemic stroke	Risk	No
**He et al, 2004 (112)** **Country:** United States **Journal:** Circulation **Sponsorship:** Not reported **COI:** Not reported **Sponsor participation in data analyses:** Not reported	Coronary heart disease mortality	Risk	No
**Huang et al, 2004 (113)** **Country:** United States **Journal:** Neurology **Sponsorship:** Government **COI:** Not reported **Sponsor participation in data analyses:** Not reported	Sporadic Parkinson disease	Risk	No
**Klement et al, 2004 (114)** **Country:** Israel **Journal:** Am J Clin Nutr **Sponsorship:** Medical center **COI:** Reported as not a conflict of interest **Sponsor participation in data analyses:** Not reported	Inflammatory bowel disease	Risk	Yes
**Kovalevsky et al, 2004 (115)** **Country:** United States **Journal:** Arch Intern Med **Sponsorship:** Not reported **COI:** Reported as not a conflict of interest **Sponsor participation in data analyses:** Not reported	Recurrent pregnancy loss	Risk	No
**Levitan et al, 2004 (116)** **Country:** United States **Journal:** Arch Intern Med **Sponsorship:** Not reported **COI:** Reported as not a conflict of interest **Sponsor participation in data analyses:** Not reported	Cardiovascular disease	Risk	No
**Lovett et al, 2004 (117)** **Country:** United Kingdom **Journal:** Neurology **Sponsorship:** Not reported **COI:** Not reported **Sponsor participation in data analyses:** Not reported	Subtype of ischemic stroke	Risk	Yes
**Mitsikostas et al, 2004 (118)** **Country:** Greece **Journal:** Brain **Sponsorship:** Not reported **COI:** Not reported **Sponsor participation in data analyses:** Not reported	Headache	Risk	No
**Montanez et al, 2004 (119)** **Country:** United States **Journal:** Arch Intern Med **Sponsorship:** Not reported **COI:** Reported as not a conflict of interest **Sponsor participation in data analyses:** Not reported	Total and cardiovascular mortality and sudden death	Risk	No
**Woodbury and Houghton, 2004 (120)** **Country:** Canada **Journal:** Ostomy Wound Manage **Sponsorship:** Not reported **COI:** Not reported **Sponsor participation in data analyses:** Not reported	Pressure ulcers	Prevalence	Yes
**Bolland et al, 2005 (121)** **Country:** New Zealand **Journal:** J Clin Endocrinol Metab **Sponsorship:** Scholarship **COI:** Not reported **Sponsor participation in data analyses:** Not reported	Increased body weight	Risk	Quality criteria abstracted
**Contopoulos-Ioannidis et al, 2005 (122)** **Country:** Greece **Journal:** J Allergy Clin Immunol **Sponsorship:** Not reported **COI:** Not reported **Sponsor participation in data analyses:** Not reported	Asthma phenotypes	Risk	Quality criteria abstracted
**Dauchet et al, 2005 (123)** **Country:** France **Journal:** Neurology **Sponsorship:** Nonprofit organization, educational institute **COI:** Reported as not a conflict of interest **Sponsor participation in data analyses:** Not reported	Stroke	Risk	No
**Etminan et al, 2005 (124)** **Country:** Canada **Journal:** BMJ **Sponsorship:** Government, fellowship **COI:** Reported as not a conflict of interest **Sponsor participation in data analyses:** Not reported	Ischemic stroke	Risk	Yes
**Fazel et al, 2005 (125)** **Country:** United Kingdom **Journal:** Lancet **Sponsorship:** Nonprofit organization **COI:** Reported as not a conflict of interest **Sponsor participation in data analyses:** "The sponsors of the study had no role in study design, data collection, data analysis, data interpretation, or writing of the report. The corresponding author had full access to all the data in the study and had final responsibility for the decision to submit for publication."	Serious mental disorder	Prevalence	Quality criteria abstracted
**García-Closas et al, 2005 (126)** **Country:** United States **Journal:** Lancet **Sponsorship:** Nonprofit organization **COI:** Reported as not a conflict of interest **Sponsor participation in data analyses:** "The study sponsors had no role in the design of the study; in the collection, analysis, or interpretation of the data; or in the writing of the report. The corresponding author had full access to all the data in the study and had final responsibility for the decision to submit the paper for publication."	Bladder cancer	Risk	No
**Lee et al, 2005 (127)** **Country:** United States **Journal:** Arthritis Rheum **Sponsorship:** Government, industry **COI:** Not reported **Sponsor participation in data analyses:** Unrestricted	Systemic lupus erythematosus	Risk	No
**Lin and August, 2005 (128)** **Country:** United States **Journal:** Obstet Gynecol **Sponsorship:** Not reported **COI:** Not reported **Sponsor participation in data analyses:** Not reported	Preeclampsia	Risk	No
**McDonald et al, 2005 (129)** **Country:** Canada **Journal:** Am J Obstet Gynecol **Sponsorship:** Not reported **COI:** Not reported **Sponsor participation in data analyses:** Not reported	Perinatal outcomes	Risk	Yes
**Palmer, 2005 (130)** **Country:** United States **Journal:** Arch Gen Psychiatry **Sponsorship:** Nonprofit organization **COI:** Not reported **Sponsor participation in data analyses:** Not reported	Lifetime risk of suicide in schizophrenia	Prevalence	Quality criteria abstracted
**Sin et al, 2005 (131)** **Country:** Canada **Journal:** Chest **Sponsorship:** Nonprofit organization, educational institute **COI:** Not reported **Sponsor participation in data analyses:** Not reported	Cardiovascular mortality	Risk	Yes
**Boudville et al, 2006 (132)** **Country:** Canada **Journal:** Ann Intern Med **Sponsorship:** Government, fellowship **COI:** Reported as not a conflict of interest **Sponsor participation in data analyses:** "The study sponsors had no role in the study design; in the collection, analysis, and interpretation of data; in the writing of the report; or in the decision to submit the paper for publication."	Hypertension	Risk	No
**Clark et al, 2006 (133)** **Country:** United Kingdom **Journal:** Pediatrics **Sponsorship:** Fellowship **COI:** Not reported **Sponsor participation in data analyses:** Not reported	Fractures	Risk	Yes
**de Boer et al, 2006 (134)** **Country:** Netherlands **Journal:** Cancer **Sponsorship:** Not reported **COI:** Not reported **Sponsor participation in data analyses:** Not reported	Unemployment	Risk	Yes
**Di Castelnuovo et al, 2006 (135)** **Country:** Italy **Journal:** Arch Intern Med **Sponsorship:** Government **COI:** Not reported **Sponsor participation in data analyses:** "The sponsor of the study had no involvement in study design; data collection, analysis, or interpretation; writing of the report; or in the decision to submit the paper for publication."	Total mortality in men and women	Risk	Yes
**Flores-Mateo et al, 2006 (136)** **Country:** United States **Journal:** Am J Clin Nutr **Sponsorship:** Nonprofit organization **COI:** Reported as not a conflict of interest **Sponsor participation in data analyses:** Not reported	Coronary heart disease	Risk	Yes
**Galassi et al, 2006 (137)** **Country:** United States **Journal:** Am J Med **Sponsorship:** Government **COI:** Not reported **Sponsor participation in data analyses:** Not reported	Cardiovascular disease	Risk	Quality criteria abstracted
**Huxley et al, 2006 (138)** **Country:** Australia **Journal:** BMJ **Sponsorship:** Government, fellowship, industry **COI:** Reported as not a conflict of interest **Sponsor participation in data analyses:** Unconditional	Fatal coronary heart disease	Risk	Quality criteria abstracted
**Kahlenborn et al, 2006 (139)** **Country:** United States **Journal:** Mayo Clin Proc **Sponsorship:** Government **COI:** Not reported **Sponsor participation in data analyses:** Not reported	Premenopausal breast cancer	Risk	Quality criteria abstracted
**Larsson et al, 2006 (140)** **Country:** Sweden **Journal:** Gastroenterology **Sponsorship:** Not reported **COI:** Not reported **Sponsor participation in data analyses:** Not reported	Esophageal, gastric, and pancreatic cancer	Risk	Quality criteria abstracted
**Mahid et al, 2006 (141)** **Country:** United States **Journal:** Mayo Clin Proc **Sponsorship:** Nonprofit organization **COI:** Not reported **Sponsor participation in data analyses:** Not reported	Inflammatory bowel disease	Risk	Yes
**Owen et al, 2006 (142)** **Country:** United Kingdom **Journal:** Am J Clin Nutr **Sponsorship:** Nonprofit organization **COI:** Reported as not a conflict of interest **Sponsor participation in data analyses:** Not reported	Type 2 diabetes	Risk	Quality criteria abstracted
**Ownby et al, 2006 (143)** **Country:** United States **Journal:** Arch Gen Psychiatry **Sponsorship:** Government **COI:** Not reported **Sponsor participation in data analyses:** Not reported	Alzheimer disease	Risk	Yes
**Pavia et al, 2006 (144)** **Country:** Italy **Journal:** Am J Clin Nutr **Sponsorship:** Not reported **COI:** Reported as not a conflict of interest **Sponsor participation in data analyses:** Not reported	Oral cancer	Risk	Yes
**Riddle et al, 2006 (145)** **Country:** United States **Journal:** Am J Trop Med Hyg **Sponsorship:** Not reported **COI:** Not reported **Sponsor participation in data analyses:** Not reported	Diarrhea	Prevalence	Yes
**Rutledge et al, 2006 (146)** **Country:** United States **Journal:** J Am Coll Cardiol **Sponsorship:** Not reported **COI:** Not reported **Sponsor participation in data analyses:** Not reported	Depression	Prevalence/ risk	Quality criteria abstracted
**Smith et al, 2006 (147)** **Country:** United States **Journal:** J Am Coll Cardiol **Sponsorship:** Government **COI:** Not reported **Sponsor participation in data analyses:** Not reported	Renal impairment	Risk	Yes
**Weis et al, 2006 (148)** **Country:** United States **Journal:** Arch Ophthalmol **Sponsorship:** Government **COI:** Reported as not a conflict of interest **Sponsor participation in data analyses:** Not reported	Uveal melanoma	Risk	Quality criteria abstracted
**Williams et al, 2006 (149)** **Country:** United Kingdom **Journal:** Arch Dis Child **Sponsorship:** Nonprofit organization **COI:** Reported as not a conflict of interest **Sponsor participation in data analyses:** Not reported	Autism spectrum disorders	Prevalence/ risk	Quality criteria abstracted
**Bahekar et al, 2007 (150)** **Country:** United States **Journal:** Am Heart J **Sponsorship:** Not reported **COI:** Not reported **Sponsor participation in data analyses:** Not reported	Coronary heart disease	Risk	Yes
**Baurecht et al, 2007 (151)** **Country:** Germany **Journal:** J Allergy Clin Immunol **Sponsorship:** Government, university **COI:** Reported as a conflict of interest **Sponsor participation in data analyses:** Not reported	Atopic eczema	Risk	No
**Bellamy et al, 2007 (152)** **Country:** United Kingdom **Journal:** BMJ **Sponsorship:** Government, fellowship **COI:** Reported as not a conflict of interest **Sponsor participation in data analyses:** Not reported	Cardiovascular disease	Risk	Quality criteria abstracted
**Conde-Agudelo et al, 2007 (153)** **Country:** Colombia **Journal:** Am J Obstet Gynecol **Sponsorship:** Government **COI:** Not reported **Sponsor participation in data analyses:** "The content of the paper has not been influenced by the sponsor."	Maternal health	Risk	Yes
**Dehghan et al, 2007 (154)** **Country:** Netherlands **Journal:** Diabetes **Sponsorship:** University, government **COI:** Not reported **Sponsor participation in data analyses:** Not reported	Diabetes	Risk	No
**Eichler et al, 2007 (155)** **Country:** Switzerland **Journal:** Am Heart J **Sponsorship:** Nonprofit organization **COI:** Not reported **Sponsor participation in data analyses:** "The funding source had no influence on study design; in the collection, analysis, and interpretation of the data; in the writing of the manuscript; and in the decision to submit the manuscript for publication."	First coronary events	Risk	Yes
**Gami et al, 2007 (156)** **Country:** United States **Journal:** J Am Coll Cardiol **Sponsorship:** Not reported **COI:** Not reported **Sponsor participation in data analyses:** Not reported	Cardiovascular events and death	Risk	Yes
**Grulich et al, 2007 (157)** **Country:** Australia **Journal:** Lancet **Sponsorship:** Government, fellowship, scholarship **COI:** Reported as a conflict of interest **Sponsor participation in data analyses:** "There was no funding source for this study. All authors had access to all the data. The corresponding author had final responsibility for the decision to submit for publication."	Cancers	Risk	Yes
**Havemann et al, 2007 (158)** **Country:** United States **Journal:** Gut **Sponsorship:** Industry **COI:** Reported as a conflict of interest **Sponsor participation in data analyses:** Not reported	Asthma	Risk	Quality criteria abstracted
**Hirtz et al, 2007 (159)** **Country:** United States **Journal:** Neurology **Sponsorship:** Not reported **COI:** Reported as not a conflict of interest **Sponsor participation in data analyses:** Not reported	Common neurologic disorders	Prevalence	Yes
**Huxley et al, 2007 (160)** **Country:** Australia **Journal:** Am J Clin Nutr **Sponsorship:** Government, nonprofit organization **COI:** Reported as not a conflict of interest **Sponsor participation in data analyses:** "None of the funding sources had any role in the study design, data analysis, data interpretation, writing of the paper, or the decision to submit the paper for publication."	Ischemic heart disease	Risk	Yes
**Krishna and Kim, 2007 (161)** **Country:** United States **Journal:** J Neurosurg **Sponsorship:** Government **COI:** Not reported **Sponsor participation in data analyses:** Not reported	Risk factors for subarachnoid hemorrhage	Risk	Quality criteria abstracted
**Langan et al, 2007 (162)** **Country:** United Kingdom **Journal:** Arch Dermatol **Sponsorship:** Nonprofit organization **COI:** Reported as not a conflict of interest **Sponsor participation in data analyses:** "The sponsor had no role in the design and conduct of the study; in the collection, analysis, and interpretation of data; or in the preparation, review, or approval of the manuscript."	Eczema	Risk	Quality criteria abstracted
**Larsson and Wolk, 2007 (163)** **Country:** Sweden **Journal:** Am J Clin Nutr **Sponsorship:** Nonprofit organization **COI:** Reported as not a conflict of interest **Sponsor participation in data analyses:** Not reported	Colon and rectal cancer risk	Risk	Quality criteria abstracted
**Larsson and Wolk, 2007 (164)** **Country:** Sweden **Journal:** Gastroenterology **Sponsorship:** Nonprofit organization **COI:** Reported as not a conflict of interest **Sponsor participation in data analyses:** "The sponsor had no role in the study design or in the collection, analysis, and interpretation of the data."	Liver cancer	Risk	Quality criteria abstracted
**Liu et al, 2007 (165)** **Country:** China **Journal:** J Am Coll Cardiol **Sponsorship:** Not reported **COI:** Not reported **Sponsor participation in data analyses:** Not reported	Recurrence of atrial fibrillation after successful electrical cardioversion	Risk	Yes
**Loza and Chang, 2007 (166)** **Country:** United States **Journal:** J Allergy Clin Immunol **Sponsorship:** Government, nonprofit organization **COI:** Reported as not a conflict of interest **Sponsor participation in data analyses:** Not reported	Atopic asthma risk	Risk	Yes
**Pittas et al, 2007 (167)** **Country:** United States **Journal:** J Clin Endocrinol Metab **Sponsorship:** Government **COI:** Reported as not a conflict of interest **Sponsor participation in data analyses:** Not reported	Type 2 diabetes	Risk	No
**Polanczyk et al, 2007 (168)** **Country:** Brazil **Journal:** Am J Psychiatry **Sponsorship:** Industry, foreign grants **COI:** Not reported **Sponsor participation in data analyses:** "There was no involvement of any funding source in the study design, data collection, analysis, interpretation of data, and writing of this article or in the decision to submit the article for publication."	Attention deficit hyperactivity disorder	Prevalence	No
**Rona et al, 2007 (169)** **Country:** United Kingdom **Journal:** J Allergy Clin Immunol **Sponsorship:** Government **COI:** Reported as a conflict of interest **Sponsor participation in data analyses:** Not reported	Food allergy	Prevalence	Quality criteria abstracted
**Sarwar et al, 2007 (170)** **Country:** United Kingdom **Journal:** Circulation **Sponsorship:** Government, scholarship, industry **COI:** Reported as not a conflict of interest **Sponsor participation in data analyses:** Unrestricted	Coronary heart disease	Risk	No
**Snoep et al, 2007 (171)** **Country:** Netherlands **Journal:** Am Heart J **Sponsorship:** Not reported **COI:** Not reported **Sponsor participation in data analyses:** Not reported	Clopidogrel nonresponsiveness	Prevalence	Yes
**Zintzaras and Kaditis, 2007 (172)** **Country:** Greece **Journal:** Arch Pediatr Adolesc Med **Sponsorship:** Not reported **COI:** Not reported **Sponsor participation in data analyses:** Not reported	Blood pressure	Risk	Yes
**Ageno et al, 2008 (173)** **Country:** Italy **Journal:** Circulation **Sponsorship:** Not reported **COI:** Not reported **Sponsor participation in data analyses:** Not reported	Venous thromboembolism	Risk	Yes
**Barclay et al, 2008 (174)** **Country:** Australia **Journal:** Am J Clin Nutr **Sponsorship:** Government **COI:** Reported as not a conflict of interest **Sponsor participation in data analyses:** Not reported	Chronic disease risk	Risk	Quality criteria abstracted
**Conde-Agudelo et al, 2008 (175)** **Country:** United States **Journal:** Am J Obstet Gynecol **Sponsorship:** Government **COI:** Not reported **Sponsor participation in data analyses:** "The views expressed in this document are solely the responsibility of the authors and do not necessarily represent the views of the World Health Organization."	Risk of preeclampsia	Risk	Yes
**Schunkert et al, 2008 (176)** **Country:** Germany **Journal:** Circulation **Sponsorship:** Government **COI:** Reported as not a conflict of interest **Sponsor participation in data analyses:** Not reported	Coronary artery disease	Risk	No
